# A Treatise on Diseases of the Nose and Throat

**Published:** 1900-12

**Authors:** 


### Book Reviews.

A TREATISE ON DISEASES OF THE NOSE AND THROAT. By ERNEST L. SHURLY, M. D, Vice-president and Professor of Laryngology and Clinical Medicine, Detroit College of Medicine; Laryngologist and late Chief of Staff, Harper Hospital; Consulting Laryngologist, St. Mary's Hospital, etc. Octavo, pp. xvii.—744. Illustrated. New York: D. Appleton & Company. 1900.

Numerous text-books on diseases of the nose and throat have appeared in the past few years purporting to be written for, the student and medical practitioner unacquainted with the manifestations of disease in the upper air passages and unfamiliar with the technique necessary for their observation. None of them has been satisfactory when measured by its purpose. Either too much knowledge on the part of the reader is assumed, or details of local surgical treatment that none but an expert is interested in or able to follow, are extended, while the broader clinical and therapeutic measures, that are of most importance and interest to the general practitioner, receive limited attention. This is, perhaps, inevitable when the point of view of the writer is habitually that of the special surgeon. It is with pleasure, therefore, that the reviewer commends Dr. Shurly's work to the student for whom it has been prepared; at the same time the most specialised of laryngologists will find broad vistas and interesting sidelights upon many points perhaps heretofore too narrowly considered.

The author is especially fitted to write such a work as he has presented to the profession. An accomplished and experienced laryngologist, he has not entirely severed himself from general practice; and he brings to the consideration of a relatively narrow field of medicine the broad views and wide general clinical experience that are constantly shown in his text-book, directly and by allusion. To one characteristic of this book it may not be amiss to call attention in these times of much false "patriotism" and "Americanism." While the author depends often, of necessity, upon reports and conclusions of foreign pathologists and experimenters, especially the Germans, he never fails to make use where possible of the original work of his fellow-countrymen, and one closes the book with a feeling almost of surprise at the fund of pathological investigations and clinical reports by Americans to which he refers.

The arrangement of subjects upon the basis of the diseased processes, rather than the anatomical relations, aids the student to a conception of the unity of the type of disease wherever manifested and prevents useless repetition. Throughout the work the clearness with which both local and general medicinal treatment are described, indicates the author's familiarity with the local and general clinical aspects of the diseases under consideration. His theories of therapeutics are clear and decided, yet practical in their application, and free from an exaggerated estimate of the value of drugs.

The first chapter of the book, devoted to the anatomy and physiology of the upper-air passages, is clear and fresh in its treatment. The illustrations are largely original, well executed, and explanatory of the features they are intended to portray. In considering the action of the soft palate, by a strange oversight no mention is made of its important function of dilating the Eustachean tube and ventilating the middle-ear. The author's lapse is the more to be regretted since, for an appreciation of the deleterious effects upon the middle-ear of lymphoid hypertrophies in the vault of the pharynx, a clear conception of this mechanism is necessary.

That portion of the work which concludes with the eleventh chapter is of particular interest to the general practitioner. In it are included the diseases of the upper-air passages which are not essentially surgical. The excellent discussion of the etiology of acute rhinitis illustrates the thoroughness and breadth with which the general relations of these local lesions are considered. If we accept the etiological relation of the bacillus of Pfeiffer to influenza, it renders more probable the microbic origin of acute rhinitis than the author implies. His trustworthy conservatism is shown, however, by his unwillingness to assume etiological factors in this and other diseases that have not satisfactory clinical as well as theoretical probability. The chapter on diphtheria,—a theme that may be called almost hackneyed,—is notable for its beautiful consideration of the pathology of the disease and for the succinctness, clearness of statement, and wisdom, of its suggestions and conclusions. It is unnecessary to add that treatment has full consideration without undue detail of obsolete methods.

In considering acute follicular tonsilitis, Dr. Shurly writes, page 139, "while it can not be denied that rheumatism is probably an etiologic factor in some instances, yet its importance in this direction has undoubtedly been very much overestimated. Whether rheumatism be considered one of the infectious diseases (due to a microörganism) or simply a hemic disease depending upon irregularities of metabolism, it has only an incidental relation, from a clinical point of view, with acute follicular tonsilitis." Again, page 143, he says: "many writers emphasise the rheumatic and gouty diathesis, as strong predisposing causes of not only this, but of the other acute and subacute diseases of the throat. It is believed that peritonsilitis has been more especially connected with the rheumatic diathesis than with any other. . . . The writer does not understand how it can be conceded on general principles that any special connection exists between the rheumatic diathesis and acute peritonsilar inflammation (quinsy) any more than between the rheumatic diathesis and other acute diseases, such as pneumonia, etc. . . Concerning the point of direct alternation or metastasis, so to speak, between rheumatic affections and acute tonsilar and other acute throat affections, all that can be said is that events of that kind are too few to furnish any basis for correct generalisation. The writer in over twenty-five years of practice, has seen only one case of acute articular rheumatism directly connected with acute peritonsilitis." Weight must be given to the author's clinical observations, broader than those of most laryngologists, but the reports of F. A. Packard, and others, of cases of endocarditis, arthritis, pleurisy, etc., following streptococcus infection of the tonsil are too detailed and suggestive to be dismissed without further investigation. This interesting change in point of view is to be noted, that while the author and the authorities he quotes have regarded the tonsilar as secondary to the rheumatic affection, the observations of Packard and others signify the dependence of the rheumatic manifestations upon microbic infection through the tonsils. No hint of this relation is found in Dr. Shurly's book.

Retropharyngeal abscess, which though rare, may yet have fatal occurrence in the practice of any family physician, receives thorough consideration. No mention is made of possible danger in using the mouth-gag in examining, or treating these cases. There is some evidence to the effect that sudden stoppage of respiration and heart may follow forcible depression of the tongue, where the abscess is located low in the pharynx.

The neuroses have full and broad treatment. Particularly may be mentioned the excellent summary of our knowledge of hay-fever, imaginary foreign-body in the pharynx, and of hysterical aphonia, each of which is frequently met with in general practice. The author speaks with special authority upon tuberculosis of the upper air-passages. The breadth and moderation of his views of the pathology of the disease will commend them to his readers. His suggestions for treatment are based on large experience and are free from undue optimism or insistence upon any special, local, or general methods of treatment.

A consideration of catarrhal inflammation of the nose, pharynx and larynx, and of diseases of the lymphoid structures of the pharynx, concludes what may be considered the medical portion of the work as distinguished from the more strictly technical and surgical part.

In the treatment of hypertrophic rhinitis no mention is made of the ingenious electrode devised by Vulpins for the removal of posterior hypertrophies of the inferior turbinal. It is so simple in its application and so satisfactory in its results that it should be more widely known and used than it appears to be. While the details of the technique of cauterisation of the nasal mucous membrane are fully given, the implication seems plain that cauterisation should not be used except where other surgical methods are contra-indicated. As to the etiology of atrophic rhinitis and ozena, it seems that scarcely enough consideration is given by the author to Grünwald's conclusions in regard to the secondary relation of this disease to suppuration of the accessory sinuses. Grünwald presents such careful study,—bibliographical, anatomical and clinical,—of these cases, and is so logical in his methods, that he compels a careful consideration of them from the point of view that he advocates. The treatment of this intractable disease is given with great fulness, but with a lack of definiteness inevitable to the present state of our knowledge.

With the author's advice that "both tonsils should not be cut at the same time" and that "only the projecting portion" should be removed the reviewer must take issue. The one procedure compels the suffering twice of post-operative sore throat,—the most painful feature of the operation; the other will result frequently in failure to remove diseased and irritating tissue. As a preventive of hemorrhage after tonsilotomy, the author does not mention the value of rest in the recumbent position until the next day. Dr. Shurly wisely avoids dogmatism about the etiology of "adenoids," but he might have laid more tress upon recurring "earache" as a symptom of the disease in childhood. The family physician still needs to be educated to appreciate that this symptom calls for prompt surgical interference, if possibly serious aural disease is to be avoided later in life. The reviewer cannot agree that "there is little danger in the administration of chloroform" in the adenoid operation. Statistics point too plainly to an excessive death-rate in this operation under chloroform. Dr. Shurly's breadth of view and conservatism in discussing the etiology of chronic laryngeal catarrh, are in contrast to the dogmatism with which narrow views of its local origin frequently have been promulgated in text-books of diseases of the upper air-tract.

The author asserts, page 498, that "nasal polypi, . . lead to chronic sinusitis of the ethmoid cells, and frontal sinus." In regard to the etiology of chronic diseases of the ethmoid cells he says, page 499, they "arise in a majority of cases from long-continued rhinitis or polypi." He does not allude to the opinion which Grünwald so ably supports in his work on Nasal Suppuration,—that inflammation of the accessory sinuses precedes and causes the polypi. Dr. Shurly, indeed, says, page 500: "Grünwald and others seem to think that nearly all cases of necrosing ethmoiditis are connected with the presence of polypi." This somewhat ambiguous statement hardly can be said to represent clearly Grünwald's position. But the whole subject of sinusitis still presents vexed questions, and the author with his usual conservatism says on the same page, "the causal relation of nasal neoplasms is possibly overrated." Perforation of the nasal wall of the antrum for diagnostic or therapeutic purposes is not usually, as stated by the author, "a very painful and often difficult procedure," whereas puncture of the nasal wall through the middle meatus with Krause's curved trochar may, as in a case known to the reviewer, penetrate the ocular orbit. In the treatment of acute sinusitis there is no mention made of the value of a free use of a spray of suprarenal extract to relieve engorgement of the mucous membrane and favor drainage. The surgical treatment of chronic sinusitis and empyema has concise, yet clear consideration. An excellent anatomical suggestion is made to the effect that "uncapping" the middle turbinate does not usually open the anterior ethmoidal cells, and that the chief value of this operation is to clear the field for access to them later. The important subject of the surgical treatment of empyema of the frontal sinus is dismissed with undue brevity. The method described by the author of opening this sinus from the inside of the nasal cavity must be condemned. The anatomical relations of the sinus, infundibulum, and cranial cavity, vary so greatly that this method of operating is much more hazardous than the external operation. Nor where granulations, necrosis, or polyps, are present in the sinus can the internal method be so thorough and satisfactory in its results.

The general practitioner will do well to heed the author's warning in cases of foreign body in the nasal passages, not to be too vigorous in his attempts at removal, lest, as in the case of a foreign body in the auditory canal, the "cure be worse than the disease." The cuts illustrative of intubation of the larynx are instructive, and the entire chapter on operations upon the pharynx and larynx repays careful reading.

Dr. Shurly's work is recommended heartily to students and general practitioners of medicine. Surgeons familiar with the field it covers will recognise its merits without assistance. Such good wine needs no bush. Praise is merited by the publishers for the handsome book they have presented to the profession; the binding, paper, type, and the execution of the illustrations are worthy of the high character of its contents.—F. W. H.

